# Measurement accuracy and reliability of self-reported versus measured weight and height among adults in Malaysia: Findings from a nationwide blood pressure screening programme

**DOI:** 10.1371/journal.pone.0280483

**Published:** 2023-01-17

**Authors:** Yook Chin Chia, Siew Mooi Ching, Pei Boon Ooi, Hooi Chin Beh, Ming Tsuey Chew, Felicia Fei Lei Chung, Navin Kumar, Hooi Min Lim

**Affiliations:** 1 Department of Medical Science, School of Medical and Life Sciences, Sunway University, Petaling Jaya, Malaysia; 2 Department of Primary Care Medicine, Faculty of Medicine, University of Malaya, Kuala Lumpur, Malaysia; 3 Department of Family Medicine, Faculty of Medicine & Health Sciences, Universiti Putra Malaysia, Kajang, Selangor, Malaysia; 4 Malaysian Research Institute on Ageing, Universiti Putra Malaysia, Serdang, Selangor, Malaysia; 5 Centre for Research, Bharath Institute of Higher Education and Research, Selaiyur, Chennai, Tamil Nadu, India; 6 Department of Primary Care Medicine, University Malaya Medical Centre, Kuala Lumpur, Malaysia; 7 Research Centre for Applied Physics and Radiation Technologies, School of Engineering and Technology, Sunway University, Petaling Jaya, Malaysia; Kyung Hee University School of Medicine, REPUBLIC OF KOREA

## Abstract

Most studies reporting prevalence of obesity use actual weight and height measurements. Self-reported weight and height have been used in epidemiological studies as they have been shown to be reliable, convenient, and inexpensive alternatives to actual measurements. However, the accuracy of self-reported weight and height might vary in different regions because of the difference in health awareness and social influences. This study aims to determine the accuracy and reliability of self-reported weight and height compared to actual measured weight and height among adults in Malaysia. This was a cross-sectional study conducted at the community level during blood pressure screening campaigns. Participants self-reported their weight and height in a questionnaire survey. Their weight and height were validated using measurements by researchers on the same setting. Body mass index (BMI) was defined as underweight (<18.5kg/m^2^), normal (18.5–22.9 kg/m^2^), overweight (23–27.4 kg/m^2^) and obesity (≥27.5 kg/m^2^). Bland-Altman analysis, intraclass correlation coefficients and weighted Kappa statistics were used to assess the degree of agreement between self-reported and measured weight and height. A total of 2781 participants were recruited in this study. The difference between the mean self-reported and measured weight and height were 0.4 kg and 0.4 cm respectively. Weighted Kappa statistics analysis showed that there was a substantial agreement between the BMI classifications derived from self-reported and actual measurement (ҡ = 0.920, p<0.001). There was no marked difference in the sensitivity and specificity of self-reported BMI among Malaysian adults by gender. We observed substantial agreement between self-reported and measured body weight and height within a sample of Malaysian adults. While self-reported body weight showed weaker agreement with actual measurements particularly for obese and overweight individuals, BMI values derived from self-reported weight and height were accurate for 88.53% of the participants. We thus conclude that self-reported height and weight measures may be useful for tracking and estimating population trends amongst Malaysian adults.

## Introduction

Anthropometric measurements such as body weight and height are important determinants for health and are usually gathered in clinical or in community settings. Most epidemiological as well as national heath and morbidity studies reporting prevalence of obesity use actual measured weight and height to drive the body mass index. However, to obtain these anthropometric parameters, actual measurements need to be conducted by trained staff using validated measuring equipment and when involving large numbers of subjects can be labour intensive and costly. Many epidemiological studies may have used self-reported methods to capture the data on weight and height in place of measured weight and height and some have shown good validity and reliability of self-reported weight and height while other studies reported conflicting results [1–3]. A literature review on the accuracy of self-reported anthropometrics showed that individuals tend to over-report their height and under-report their weight for both genders [4]. The discrepancy between self-reporting and actual measurement was greater among overweight and obese adults [5, 6] and in those who were underweight [7]. Mixed results in the accuracy of self-reported weight and height in different countries were also seen and attributed to the differences in health awareness, culture, and social influences [8]. Asian populations showed a smaller difference between self-reported and measured weight and height compared to North American populations [4]. In Malaysia, the country with the highest prevalence of overweight and obesity in South-East Asia, overweight and obesity are major contributors to the burden of disease [9]. As self-reported weight and height measurements are used in many epidemiological studies as a measure of overweight/obesity and BMI category, determining the accuracy and reliability of self-reported heights and weight is important [10]. Self-reporting bias may result in inaccuracies in the classification of the nutritional status and thus leads to unreliable estimation of the prevalence of overweight/obesity in a community. Self-reported measurements are easier, less costly and more convenient than actual measurement and are particularly useful in dissemination especially to track and monitor changes in prevalence obesity over time [11]. It is thus critical that population-specific assessments on the reliability of self-reported data are conducted to determine the extent of the biases that may be introduced when using self-reported anthropometric measures and to identify demographic factors affecting the reliability of self-reported information. However, there are limited studies reporting the accuracy and reliability of self-reported weight and height among adults in Asia [2, 12]. Hence, our study aims to determine the measurement accuracy and test-retest of self-report weight and height among adults in Malaysia.

## Methods

Ethics approval was obtained from the National Medical Research Register (NMRR-18-876-40691) and University of Malaya Medical Centre-Medical Research Ethics Committee (MREC ID NO:2018320–6146). This paper is part of a cross-sectional study conducted at the community level during worldwide BP screening campaign [13, 14]. The methodology has been described in a previous publication on the main [15] and Malaysia study [16]. In brief, the duration of the study was from the 1^st^ of May to the 31^st^ of October 2018. The screening programme was carried out at 22 centres throughout east and west Malaysia using a standardized protocol by 25 site investigators. A total of 5172 individuals participated in this campaign with 2781 (53.8%) providing self-reported measures of their height and weight.

### Sample size calculation

The sample size was not calculated as this was a public screening programme. A similar study conducted among Malaysian adolescents reported a sample size of 663 and weighted Kappa statistics reported a substantial agreement between BMI status based on self-reported weight and height and the actual measurements (kappa = 0.76, 95% CI: 0.67, 0.84) [2]. Our sample size of 2781 is powered enough to test the concordance between measured and self-reported weights and heights.

### Sampling and data collection

All individuals aged 18 years and older who came forward to be screened for hypertension were eligible for this study. We excluded those with presence of psychiatric illness. The blood pressure screening campaign was conducted in the community. Researchers explained the study to participants, and verbal consent was obtained prior to the study. We included in this analysis all participants who had both measured and reported weight and height.

A self-administered questionnaire was used to obtain socio-demographic (age, ethnicity, education level, and occupations) data. Participants were also asked to report their own height and weight. Subsequently, participants’ height and weight were measured by trained researchers.

### Measurements

Weight and height measurements were performed by the researchers according to the standard procedures. The weight was measured and recorded to the nearest 0.1 kg, using Rossmax WB 101 scale. Weights were recorded in kilograms (kg) while heights were recorded in centimetres (cm). Participants were informed to remove their footwear, heavy outer garments, and any heavy items from their pockets. Participant height was measured using Seca scale. Participants were told to stand with their feet flat on the floor and with the back of their head, shoulder, and buttocks against the Seca scale. Participants were asked to stand straight and to look forward. The researchers lowered the measuring indicator until it rested on top of the head, and recorded the measured body height to the nearest 0.1 centimetres (cm). BMI was calculated as weight (in kilograms) divided by height (in meters) squared.

### BMI classification

The Asian criteria for classifying the subjects’ BMI was used in our study as Asians tend to manifest weight-related diseases such as diabetes mellitus or hypertension at lower BMIs than in western populations [17]. The Asian criteria defines Underweight as BMI <18.5kg/m [18], Normal weight as BMI between 18.5 to 22.9 kg/m^2^, Overweight as BMI between 23 to 27.4 kg/m^2^, and obesity as BMI ≥27.5kg/m^2^ [17].

### Underreporting and over-reporting of body weight and height

Underreporting of body weight and height were defined as those measurements of self-reported were more than 5% below the actual measurement for weight and height. Overreporting was defined as those measurements of self-reported more than 5% above the actual measurement for weight and height [19].

### Statistical analysis

Descriptive statistics were used to analyse the socio-demographics data. Agreement between measured and self-reported measures of height and weight was assessed using Bland-Altman analyses [20], intra-class correlation coefficients [21], and quadratic weighted Kappa statistics [22] as described previously.

Briefly, Bland-Altman analyses were performed to examine the degree of agreement between self-reported and measured weight and height [21]. In a Bland-Altman plot, the discrepancy between two measurements was plotted against the mean of the two measured values. The horizontal lines were drawn at the mean difference and at the upper and lower limits of the 95% confidence interval (CI) of agreement. The upper and lower limits of agreement were calculated as the mean difference between self-reported and measured parameters ±1.96 x (standard deviation of the mean difference) based on one independent t-test [20]. The mean difference indicated the degree of bias between self-reported and measured values. The 95% CI of agreement demonstrates the precision of the mean difference which implies how far apart the self-reported weight, height, and BMI were from the actual measurement. The limits of agreement derived from the Bland-Altman analyses were then compared to the limits of over- and under-reporting of body height and weight described in a previous section (5% in excess or below actual measurements).

The Intraclass Correlation Coefficient (ICC) was used to derive a summary measure of absolute agreement between self-reported and measured weight and height in this study [23]. The ICC ranges from 0 (no agreement) to 1 (perfect agreement), where ICC of 0.75 to 0.9 indicates good reliability [24]. Weighted Kappa statistics were used to examine the degree of agreement between BMI categorization derived from self-reported values and BMI categorization derived from actual measurements [22]. The BMI was classified into underweight, normal weight, overweight, and obesity by gender. A Kappa (κ) of 0.80 and above represents a strong strength of agreement [25, 26]. We calculated the sensitivity, specificity, positive predictive value (PPV), and negative predictive value (NPV) of self-reported BMI status with the gold standard measured BMI.

Descriptive statistics were performed using SPSS version 23 and a *p*-value <0.05 was considered to be statistically significant. Blant-Altman analyses, intraclass correlations, and Fleiss-Cohen Kappa computations were conducted using the BlandAltmanLeh (v. 0.3.1), irr (v. 0.84.1), and vcd (v. 1.4–8) R packages respectively. Bland-Altman plots were visualized using the ggplot2 (v. 3.3.3) packages in R 4.0.3.

## Results

A total of 2781 participants provided self-reports of their height and weight. The median age of the participants was 32 years. Most of the participants were female (59.9%) and identified their ethnicity as Malay (60.2%). Most of the participants had a tertiary level of education (n = 1722, 62.3%). Additional information on participants’ demographics is provided in Table 1.

**Table 1:** Sociodemographic characteristics of the study participants (n = 2781).

Variables	n (%)	
Age (Median, IQR) years		32 (28)
Gender (n = 2778)		
Male	1113 (40.1)	
Female	1665 (59.9)	
Ethnicity (n = 2772)		
Malay	1668(60.2)	
Chinese	671 (24.2)	
Indian	247 (8.9)	
Others	186 (6.7)	
Occupation (n = 2754)		
Retired	298 (10.9)	
Housewife	692 (25.1)	
College /University	604 (21.9)	
Skilled Worker	449 (16.3)	
Unskilled Worker	298 (10.8)	
Professional Worker	413 (15.0)	
Highest Education (n = 2763)		
None	43 (1.6)	
Primary	157 (5.7)	
Secondary	841 (30.4)	
Tertiary	1722 (62.3)	

The majority (61.7%) of the participants were overweight or obese, which is higher than the reported national average of 50.1%. The proportion of overweight or obese respondents was higher in male respondents (66.4%) than in females (58.6%) (Table 2). The overall mean values of measured and self-reported height were virtually identical, at 161.3 ± 8.8 cm and 160.9 ± 8.8 cm respectively. The same was observed with measured and self-reported body weight, which were 65.6 ± 15.6 kg and 65.2 ± 15.2 kg respectively and for BMI (25.2 ± 5.5 kg/m^2^ and 25.2 ± 5.6 kg/m^2^ respectively) (Table 2).

**Table 2:** Agreement between measured and self-reported values for height and weight in all participants, and stratified by genders and BMI category.

			Height (cm)			Weight (kg)			BMI (kg/m^2^)		
			Measured	Self-reported	Intraclass correlation coefficient	Measured	Self-reported	Intraclass correlation coefficient	Measured	Self-reported	Intraclass correlation coefficient
		Counts, n (%)	Mean (SD)	Mean (SD)		Mean (SD)	Mean (SD)		Mean (SD)	Mean (SD)	
All participant		2781 (100)	161.3 (8.8)	160.9 (8.8)	0.9319	65.6 (15.6)	65.2 (15.2)	0.9605	25.2 (5.5)	25.2 (5.6)	0.9289
Gender	Male	1113 (40.1)	166.8 (8.4)	166.3 (8.5)	0.9255	70.7 (16.4)	70.1 (15.7)	0.9262	25.4 (5.6)	25.3 (5.4)	0.9091
	Female	1665 (59.9)	157.6 (7.0)	157.3 (7.1)	0.8920	62.3 (14.1)	61.9 (14)	0.9852	25.1 (5.5)	25.1 (5.7)	0.9416
BMI category, (All participant)	Underweight	188 (6.8)	162.5 (9)	161.5 (8.6)	0.9091	45.4 (5.4)	45.4 (5.9)	0.9258	17.2 (1.1)	17.4 (1.7)	0.6261
	Normal	878 (31.6)	161.5 (8.3)	161 (8.4)	0.9531	54.8 (6.6)	54.8 (7.1)	0.9289	21 (1.3)	21.1 (1.7)	0.6987
	Overweight	940 (33.8)	161.6 (8.9)	161.3 (8.9)	0.9319	65.9 (8)	65.5 (8.9)	0.8590	25.2 (1.3)	25.1 (2.1)	0.4726
	Obese	775 (27.9)	160.4 (9.1)	160.3 (9.4)	0.9168	82.5 (14.9)	81.3 (13.8)	0.9178	32 (4.8)	31.7 (5.2)	0.8008
BMI category, (males)	Underweight	74 (6.6)	167.3 (9.2)	166.2 (8.9)	0.8746	47.9 (5.5)	48.2 (6.3)	0.8809	17.1 (1.3)	17.5 (2.1)	0.5406
	Normal	300 (27)	167.2 (8.5)	166.7 (8.7)	0.9447	58.9 (6.9)	59.1 (8)	0.8969	21 (1.3)	21.2 (1.9)	0.6475
	Overweight	428 (38.5)	166.6 (8.2)	166.2 (8.2)	0.9229	69.9 (7.7)	69.4 (9.5)	0.7604	25.1 (1.2)	25.1 (2.2)	0.3466
	Obese	311 (27.9)	166.7 (8.3)	166.2 (8.8)	0.9243	88.5 (15.9)	86.7 (13.5)	0.8306	31.9 (5.5)	31.5 (5)	0.7946
BMI category, (females)	Underweight	113 (6.8)	159.3 (7.4)	158.5 (7)	0.9025	43.8 (4.8)	43.7 (4.7)	0.9789	17.2 (1.1)	17.4 (1.3)	0.8077
	Normal	576 (34.6)	158.5 (6.5)	158.1 (6.6)	0.9322	52.6 (5.3)	52.5 (5.4)	0.9255	20.9 (1.3)	21 (1.6)	0.7321
	Overweight	512 (30.8)	157.5 (7.1)	157.1 (7.1)	0.8912	62.6 (6.5)	62.2 (6.9)	0.9192	25.2 (1.3)	25.2 (1.9)	0.5984
	Obese	464 (27.9)	156.2 (7.1)	156.3 (7.6)	0.8455	78.5 (12.7)	77.7 (12.9)	0.9776	32.1 (4.4)	31.8 (5.4)	0.8057

Overall, we observed excellent agreement between measured and self-reported values for height, weight, and the derived BMI, where ICC values obtained are 0.9319, 0.9605, and 0.9289 respectively using the unstratified dataset. ICC values were greater than 0.9 for height, weight, and BMI when stratified by gender, with the exception of height measures among females (0.8920). When stratified by BMI category, ICC values exceeded 0.75 for height and weight measures, considered a “good” level of agreement in Koo and Li (2016), with the lowest agreement being observed in overweight males (0.7604). Despite the generally high level of agreement in height and weight measures among all subgroups when assessed by ICC (> 0.75), relatively low levels of agreement were observed between BMI calculated from measured and self-reported heights and weights. Agreement was lowest for BMI values in the overweight group as whole (0.4726). When stratified by both BMI category and gender, agreement was lowest in overweight males (0.3466), followed by underweight males (0.5406), and overweight females (0.5984).

Differences between actual and self-reported height and weight values led to the classification of 319 (11.5%) of the participants into the incorrect BMI category. Nevertheless, assessments of the agreement between BMI values derived from self-reported and measured height and weight values by weighted Kappa indicate that there is a statistically significant (p < 0.001) and good agreement between the two sets of derived BMIs for all participants (κ = 0.920), males (κ = 0.895), and females (κ = 0.935) (Table 3).

**Table 3:** Comparison of BMI categories derived from self-reported and measured height and weight values in all participants, and when with stratification by gender.

BMI category derived from self-reported height and weight (n)	BMI category derived from measured height and weight (n, %)				
	Underweight (BMI<18.5kg/m2)	Normal (BMI 18.5–22.9kg/m2)	Overweight (BMI 23–27.4kg/m2)	Obese (BMI>27.5kg/m2)	Totals in self-reported category
*All participant*					
Underweight (BMI<18.5kg/m2)	166 (88.30)	25 (2.85)	4 (0.43)	0 (0.00)	195 (7.01)
Normal (BMI 18.5–22.9kg/m2)	20 (10.64)	783 (89.18)	62 (6.60)	4 (0.52)	869 (31.25)
Overweight (BMI 23–27.4kg/m2)	1 (0.53)	65 (7.40)	822 (87.45)	80 (10.32)	968 (34.81)
Obese (BMI ≥ 27.5kg/m2)	1 (0.53)	5 (0.57)	52 (5.53)	691 (89.16)	749 (26.93)
Totals in measured category	188 (100.00)	878 (100.00)	940 (100.00)	775 (100.00)	2781 (100)
Fleiss-Cohen Kappa statistic (95% CI)	**0.920 (0.910–0.931)**				
*Males*					
Underweight (BMI<18.5kg/m2)	62 (83.78)	7 (2.33)	4 (0.93)	0 (0)	73 (6.56)
Normal (BMI 18.5–22.9kg/m2)	11 (14.86)	258 (86)	33 (7.71)	2 (0.64)	304 (27.31)
Overweight (BMI 23–27.4kg/m2)	0 (0)	33 (11)	363 (84.81)	33 (10.61)	429 (38.54)
Obese (BMI ≥ 27.5kg/m2)	1 (1.35)	2 (0.67)	28 (6.54)	276 (88.75)	307 (27.58)
Self-reported totals	74 (100)	300 (100)	428 (100)	311 (100)	1113 (100)
Fleiss-Cohen Kappa statistic (95% CI)	**0.895 (0.875–0.916)**				
*Females*					
Underweight (BMI<18.5kg/m2)	103 (91.15)	18 (3.13)	0 (0)	0 (0)	121 (7.27)
Normal (BMI 18.5–22.9kg/m2)	9 (7.96)	523 (90.8)	29 (5.66)	2 (0.43)	563 (33.81)
Overweight (BMI 23–27.4kg/m2)	1 (0.88)	32 (5.56)	459 (89.65)	47 (10.13)	539 (32.37)
Obese (BMI>/ 27.5kg/m2)	0 (0)	3 (0.52)	24 (4.69)	415 (89.44)	442 (26.55)
Self-reported totals	113 (100)	576 (100)	512 (100)	464 (100)	1665 (100)
Fleiss-Cohen Kappa statistic (95% CI)	**0.935 (0.924–0.946)**				

Significant *p*-values (p < 0.001) shown in bold text.

To gain a better understanding of the directionality and magnitude of differences between self-reported and measured body height and weight, mean and standard deviation were calculated for the differences in self-reported and measured values.

Based on these definitions, the large majority of participants provided accurate measures of their body height (>90% of participants in all categories) and weight (>80% of participants in all categories) (Table 4). Overall, the majority of participants (96.51%) accurately reported their height and 89% correctly reported their weight. Mean differences between self-reported and measured height and weight values tended to be negative, i.e., participants tended to underestimate their body height and weight, albeit within an acceptable range. 1.19% (n = 33) participants over-reported their heights by an average difference of 11.79 ± 3.52, and 2.3% (n = 64) underreported their heights by an average of 13.89 ± 7.71 cm. 3.25% (n = 91) of the participants overreported their weights with an average overestimation of 9.15 ± 10.4 kg, while 7.73% (n = 215) underestimated their body weight at an average of 7.50 ± 9.64 kg.

**Table 4:** Underreporting^a^ and overreporting^b^ of body weight and height by gender and by BMI category.

		Height (cm)						Weight (kg)					
		Accurate		Underreporting		Overreporting		Accurate		Underreporting		Overreporting	
		Counts, n (%)	Mean (SD)	Counts, n (%)	Mean (SD)	Counts, n (%)	Mean (SD)	Counts, n (%)	Mean (SD)	Counts, n (%)	Mean (SD)	Counts, n (%)	Mean (SD)
All participants		2684 (96.51)	-0.2 (1.78)	64 (2.30)	-13.89 (7.71)	33 (1.19)	11.79 (3.52)	2475 (89.00)	-0.23 (1.06)	215 (7.73)	-7.5 (9.64)	91 (3.27)	9.15 (10.4)
Gender (All participants)	Male	1067 (95.87)	-0.24 (2.01)	34 (3.05)	-12.28 (4.28)	12 (1.08)	11.58 (3.65)	962 (86.43)	-0.24 (1.18)	103 (9.25)	-9.56 (13.27)	48 (4.31)	10.85 (13.00)
	Female	1614 (96.94)	-0.17 (1.60)	30 (1.80)	-15.71 (10.09)	21 (1.26)	11.91 (3.53)	1511 (90.75)	-0.23 (0.98)	111 (6.67)	-5.54 (3.03)	43 (2.58)	7.26 (5.97)
BMI category (All participants)	Underweight	181 (96.28)	-0.53 (1.58)	6 (3.19)	-17 (7.72)	1 (0.53)	12 (NA)	174 (92.55)	-0.05 (0.75)	8 (4.26)	-4.98 (3.52)	6 (3.19)	7.08 (6.54)
	Normal	853 (97.15)	-0.28 (1.65)	18 (2.05)	-11.13 (2.96)	7 (0.80)	11.49 (3.81)	799 (91.00)	-0.15 (0.9)	44 (5.01)	-4.26 (1.32)	35 (3.99)	8.25 (7.65)
	Overweight	906 (96.38)	-0.21 (1.83)	22 (2.34)	-14.27 (5.84)	12 (1.28)	11.88 (3.65)	818 (87.02)	-0.23 (1.01)	85 (9.04)	-7.09 (5.58)	37 (3.94)	10.38 (14.2)
	Obese	744 (96.00)	-0.01 (1.88)	18 (2.32)	-15.14 (11.76)	13 (1.68)	11.85 (3.68)	684 (88.26)	-0.38 (1.33)	78 (10.06)	-10.04 (14.45)	13 (1.68)	9.03 (3.63)
BMI category, males	Underweight	70 (94.59)	-0.67 (1.87)	3 (4.05)	-17.33 (10.21)	1 (1.35)	12 (NA)	67 (90.54)	-0.03 (0.96)	3 (4.05)	-4.17 (1.36)	4 (5.41)	9.2 (7.30)
	Normal	288 (96.00)	-0.23 (1.83)	10 (3.33)	-10.35 (1.42)	2 (0.67)	13.35 (4.74)	268 (89.33)	-0.07 (0.98)	16 (5.33)	-4.83 (1.67)	16 (5.33)	9.66 (9.27)
	Overweight	410 (95.79)	-0.33 (2.04)	10 (2.34)	-12.27 (3.55)	8 (1.87)	11.28 (4.04)	362 (84.58)	-0.21 (1.07)	47 (10.98)	-8.37 (6.69)	19 (4.44)	13.1 (18.61)
	Obese	299 (96.14)	-0.04 (2.17)	11 (3.54)	-12.67 (3.86)	1 (0.32)	10 (NA)	265 (85.21)	-0.5 (1.49)	37 (11.90)	-13.57 (20.25)	9 (2.89)	8.93 (3.44)
BMI category, females	Underweight	110 (97.35)	-0.44 (1.37)	3 (2.65)	-16.67 (6.66)	0 (0.00)	NA	107 (94.69)	-0.07 (0.60)	4 (3.54)	-3.5 (1)	2 (1.77)	2.85 (0.07)
	Normal	563 (97.74)	-0.31 (1.56)	8 (1.39)	-12.1 (4.09)	5 (0.87)	10.74 (3.70)	529 (91.84)	-0.19 (0.85)	28 (4.86)	-3.94 (0.97)	19 (3.30)	7.07 (5.98)
	Overweight	496 (96.88)	-0.12 (1.63)	12 (2.34)	-15.93 (6.94)	4 (0.78)	13.1 (2.81)	456 (89.06)	-0.25 (0.96)	38 (7.42)	-5.52 (3.23)	18 (3.52)	7.5 (6.57)
	Obese	445 (95.91)	0 (1.66)	7 (1.51)	-19.03 (18.39)	12 (2.59)	12.01 (3.80)	419 (90.3)	-0.3 (1.21)	41 (8.84)	-6.85 (3.30)	4 (0.86)	9.25 (4.60)

**Fig 1:**
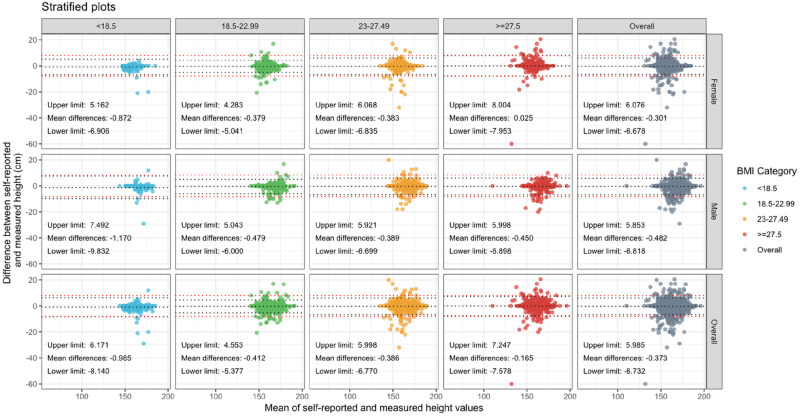
Bland-Altman plots showing the difference between self-reported and measured height against mean of self-reported and measured height by gender and BMI. Mean differences and the limits of agreement are denoted in each panel and are represented by black dotted lines. The definition of “accurate” for the purpose of this study are within 5% of mean participant height which is denoted with red dotted lines for each category.

These findings were corroborated by Bland-Altman analyses that were used to assess agreement between self-reported and measured body height and weight for all participant categories of gender and BMI. In estimations of body height, the limits of agreement, defined as the mean difference ± 1.96 standard deviations, lay within the pre-defined limits of accuracy (i.e., ± 5% of the mean measured height). This was true for all participant categories, indicating that self-reported height can be used as a reliable estimate of actual participant height in studies involving Malaysian adults (Fig 1).

**Fig 2:**
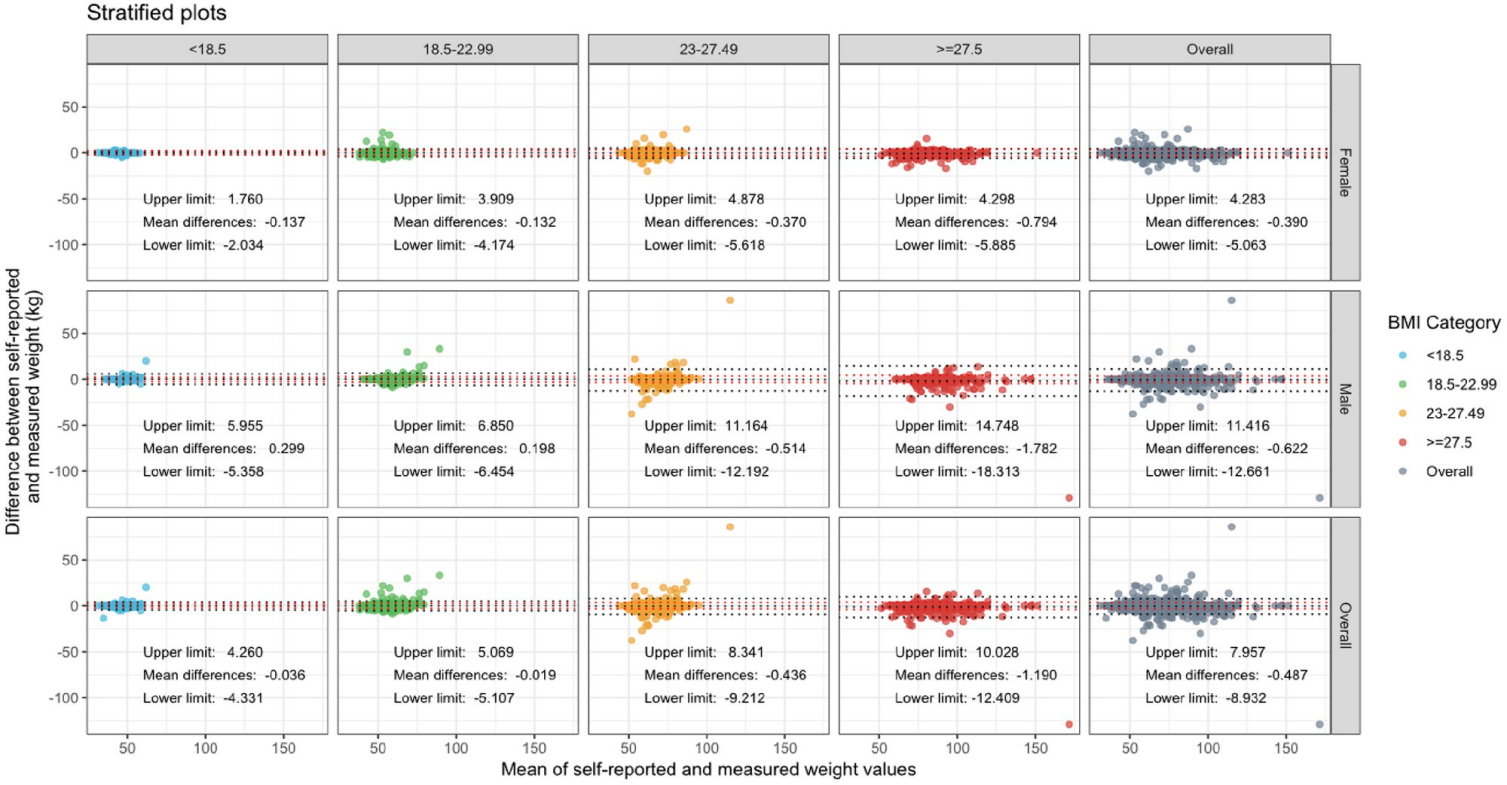
Bland-Altman plots showing the difference between self-reported and measured weight against mean of self-reported and measured weight by gender and BMI. Mean differences and the limits of agreement are denoted in each panel and are represented by black dotted lines. The definition of “accurate” predictions for the purpose of this study are within 5% of mean participant weight which is denoted with red dotted lines for each category.

Similarly, in estimations of body weight, the limits of agreement, as previously defined, were wider than the limits of accuracy defined as ± 5% of the mean measured body weight for all participant groups with the exception of underweight females. Differences were amplified in males and in overweight or obese participants (Fig 2).

Table 5 shows that there was high sensitivity (Sn) and specificity (Sp) of self-reported BMI as compared to the measured BMI; underweight, Sn 88.3% and Sp98.9%; normal weight, Sn 90.5% and Sp 95.7%; overweight, Sn 88.1% and Sp 92.9%; and obesity Sn 90.1% and Sp 97.3%. There was no marked difference in sensitivity and specificity of self-reported BMI for identifying underweight, normal weight, overweight, and obese adults by gender. The positive predictive value (PPV) of BMI-for-age for all participants for overweight and obesity was 86.3% and 92.9% respectively and the corresponding negative predictive values (NPV) for overweight and obesity were 93.9% and 96.1%.

**Table 5:** Sensitivity, specificity, positive predictive value, and negative predictive value of self-reported BMI classifications by gender.

	All				Male				Female			
	Sn (%)	Sp (%)	PPV (%)	NPV (%)	Sn (%)	Sp (%)	PPV (%)	NPV (%)	Sn (%)	Sp (%)	PPV (%)	NPV (%)
Underweight	88.3	98.9	86.4	99.1	83.8	98.9	84.9	98.8	91.2	99.0	87.3	99.3
Normal weight	90.5	95.7	90.5	95.7	87.8	94.6	85.4	95.6	91.9	96.5	93.3	95.8
Overweight	88.1	92.9	86.3	93.9	85.4	91.7	86.7	90.9	90.3	93.6	86.0	95.7
Obesity	90.1	97.3	92.9	96.1	90.2	96.3	90.5	96.2	90.0	97.9	94.5	96.1

Sn: sensitivity, Sp: specificity, PPV: positive predictive value, NPV: Negative predictive value.

## Discussion

This present study reported a strong correlation between self-reported and measured weight and height among Malaysian adults. A large majority of the participants provided accurate estimates of their weight and height (89.00% and 96.51% respectively). Interestingly, agreements between self-reported and measured body weight was weakest in overweight compared to obese participants.

BMI values derived from self-reported measures of weight and height generally agreed with that derived from actual measures of weight and height (Fleiss-Cohen Kappa statistic > 0.89 in all categories), with stronger agreement being observed in female participants compared to male participants. However, the limits of agreement for participant weight exceeded that of the acceptable range of ± 5% of the mean measured body weight, indicating that self-reported weight data should be interpreted with caution. Nevertheless, BMI categories calculated based on self-reported data was accurate for 88.53% of all participants, and thus may be adequate for monitoring population health trends.

In general, BMI has been reported to be a negative component contributing to body image [27] and the fear of judgement [28] and BMI underestimation in those overweight seems to be still persisting till today with female underreported their weight [4, 29] while some studies showed vice versa [18, 30]. We found the underreporting of weight and height in this present study did not affect the BMI classification. The underreported pattern of both variables observed in this study was similar to the previous study conducted among Malaysian adolescents [2] and Indonesians [10]. Other similar studies conducted in Denmark [31], USA [18], and Japan [32] showed good correlation between self-reported and actual measurement of height, weight, and BMI. Studies in United States showed an overestimation of height and underestimation of weight which caused discrepancy in BMI category [31]. Self-reported data were more likely to underestimate the measured data in studies conducted in women only, which is comparable to the findings in our study [29, 33]. Overestimating self-reported height and underestimating self-reported weight lead to an underestimation of BMI [4]. The different patterns of bias were likely due to the demographic characteristics of the study populations. Age, gender, and higher BMI groups were the common factors that contribute to the different and extreme results in studies [4, 34].

In our study, there was a good agreement between self-reported BMI and measured BMI with a mean difference of less than one unit. This indicates that self-reported weight and height by Malaysian adults were reliable and accurate for BMI calculation. Our findings were consistent with the study conducted among Malaysian adolescents [2]. This study highlights the accuracy and reliability of self-reported weight and height among Malaysian adults. Self-reported weight and height could be a feasible and reliable measurement especially in online or mass screening surveys in Malaysia. The accuracy of self-reported weight and height reported in our study reflects the awareness of the general population on their body weight. It is feasible for the general population to self-assess their health using some of the simple risk screening tools such as the Finnish Diabetes Risk Score [35] and Osteoporosis Self-assessment Tool for Asians [36] which require weight and height measurements.

There were several strengths in this study. Firstly, it was conducted nationwide in multiple sites (both rural and urban health and public settings) and covered a large number of participants. Second, this study followed an established international protocol in obtaining the self-reported and measured weights and heights among participants. For limitations, only 2781 participants self-reported their weight and height during the questionnaire survey among a total of 5172 participants who participated in the screening campaign. We acknowledge the possibility of selection bias here. Second, most of the participants had a tertiary educational level, and the findings may not be generalizable to the population with lower educational levels.

## Conclusions

Our study showed that self-reported weights and heights among Malaysian adults are accurate and reliable to be used as an indicator for public health-related research and data collection. Identification of obesity in individuals or at the population level can be achieved through self-reported weight and height.

## Supporting information

S1 Dataset Data set of all participants (N = 2781) with socio demographics, measured and self-reported height and weight. (XLSX)
